# Virome Structure of the Small Aral Sea

**DOI:** 10.1128/MRA.01023-20

**Published:** 2020-10-08

**Authors:** Madina Alexyuk, Andrey Bogoyavlenskiy, Makhabbat Amanbayeva, Pavel Alexyuk, Yergali Moldakhanov, Elmira Anarkulova, Anar Imangazy, Vladimir Berezin

**Affiliations:** a Research and Production Center for Microbiology and Virology, Laboratory of Antiviral Protection, Almaty, Kazakhstan; b Abai Kazakh National Pedagogical University, Almaty, Kazakhstan; DOE Joint Genome Institute

## Abstract

Here, we present a virome analysis of the surface waters of the Small Aral Sea. In this case, shotgun metagenomic sequencing of the RNA and DNA virus community was used.

## ANNOUNCEMENT

The Aral Sea is a unique relict inland lake undergoing a global ecological disaster. In the late 1980s, with the water receding, the Aral Sea was divided into the northern (Small Aral Sea) and southern (Large Aral Sea) parts (1). For a more precise description of the changing environmental conditions in the region, it is necessary to study the diversity of all types of life forms, including viruses, which are key elements of the nutrient cycle (2).

The aim of the study was to describe and analyze the viral community of the Small Aral Sea by shotgun sequencing. Three water samples were collected from the surface of the Small Aral Sea at 1-week intervals in May 2019. The coordinates of the sampling site are 46.410263N and 61.291999E (about 1 to 1.5 m deep).

The freshly collected water samples (each about 5 liters, in sterile bottles) were sequentially filtered through 3-μm and 0.22-μm filters, concentrated to 500 ml with 50-kDa cartridges (Vivaflow 200, with polyethersulfone membranes; Sartorius), pooled, and concentrated by ultracentrifugation (Avanti J30I; Beckman Coulter) at 29,000 rpm for 2 h at 4°C. The precipitate was dissolved in a minimum volume of phosphate-buffered saline (pH 7.2).

Viral RNA was extracted using a QIAamp viral RNA minikit (Qiagen). The sample extracts were pretreated with RNase-free DNase (Promega). Double-stranded cDNA was obtained with a SuperScript double-stranded cDNA synthesis kit (Invitrogen) according to the manufacturer’s instructions. Total DNA was isolated using a PureLink genomic DNA extraction kit (Thermo Fisher Scientific, USA) and stored at −80°C. Genomic DNA and synthesized double-stranded cDNA were pooled. The pooled DNA sample was used for further sequencing and library preparation.

DNA libraries were prepared from 1 ng of the obtained double-stranded DNA using the Nextera XT DNA sample preparation kit (Illumina, USA) in accordance with the manufacturer’s instructions. The libraries were sequenced using the Illumina MiSeq platform (paired-end sequencing, 2 × 300 bp; MiSeq kit v3).

The initial quality control of the sequences obtained was performed with FastQC v0.11.9 (https://www.bioinformatics.babraham.ac.uk/projects/fastqc) and Trimmomatic v0.36 (3). Starting with 3,248,602 reads, 42,241 reads (1%) that did not pass quality control were removed. The analysis of the metagenomic data obtained was performed using Geneious Prime v2020.2 and Kaiju v1.7.3 (http://kaiju.binf.ku.dk) (4–6). Default parameters were used for all software.

**FIG 1:**
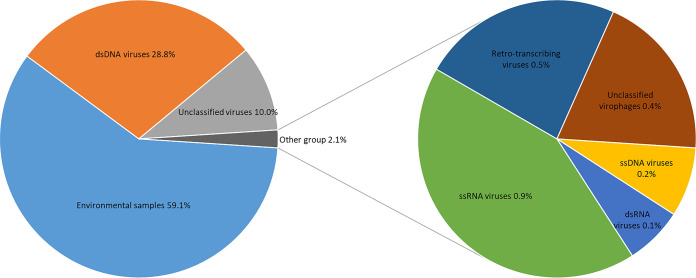
Relative distribution of reads in the virome. The distribution of the metagenomic reads was based on the NCBI BLAST nonredundant database; Microsoft Excel 2016 was used to generate the plots. ss, single-stranded; ds, double-stranded.

Taxonomic classification of the metagenomic data for the Small Aral Sea showed that 673,835 reads of the total raw sequencing data corresponded to viruses. Figure 1 represents the diversity of the viral community of the investigated sample. The virome of the Small Aral Sea included double-stranded DNA, environmental samples (uncultured marine viruses), single-stranded RNA, single-stranded DNA, double-stranded RNA, retrotranscribing viruses, unclassified viriophages, and unclassified viruses.

This study provides the first report on the viral diversity of the Small Aral Sea, which expands our knowledge of the diversity of the Aral-Syr Darya water basin.

### Data availability.

The raw sequence reads are available under BioProject accession number PRJNA643916 and SRA accession number SRR12265913.
